# Irradiation impact on biological activities of Anthraquinone pigment produced from *Talaromyces purpureogenus* and its evaluation, characterization and application in beef burger as natural preservative

**DOI:** 10.1186/s12866-022-02734-4

**Published:** 2022-12-30

**Authors:** Ibrahim A. Soliman, Yasmeen A. Hasanien, Amira G. Zaki, Hany A. Shawky, Amr A. Nassrallah

**Affiliations:** 1 Plant Research Department, Nuclear Research Center, Egyptian Atomic Energy Authority, Cairo, Egypt; 2 Biochemistry Department, Faculty of Agriculture, Cairo University, Giza, Egypt

**Keywords:** Natural pigment, Anthraquinone, *Talaromyces purpureogenus*, Food preservative, Food irradiation

## Abstract

**Background:**

The demand for natural coloring and preservative agents in food industry is increasing day by day as a result of awareness of the negative health effects of synthetic color preservatives. Consumers want foods with less processing, a longer shelf life, and clear labels that list only natural ingredients and food additives with familiar names that promote good health. In order to meet consumer demands and regain consumers' confidence in the safety of food products, the food industry was compelled to search for natural alternatives with strong antibacterial and antioxidant properties. Therefore, the objective of this study was to produce a microbial pigment that not only serve as food coloring agents but also provide health advantages owing to their bioactivities. Additionally, the potential use of anthraquinone pigment (AQP) as a natural food preservative compared to gamma irradiation was also examined to extend the shelf life of the beef burger and improve its hygienic quality.

**Results:**

This study used *Talaromyces purpureogenus* AUMC2603 to produce the red natural pigment, which was identified as an anthraquinone pigment (AQP). According to the results, gamma (γ) radiation had no significant effect on AQP's antibacterial properties. However, it has a negative, considerable effect on antioxidant activity, where a large dose of γ-ray may change the antioxidant components and lessen the AQP's capacity to scavenge free radicals. Additionally, the γ ray-treated AQP had a strong cytotoxic activity in relation to a high γ-ray dose. As a result, it is suggested that AQP-containing foods should not be irradiated. The extracted AQP was applied as a food additive to improve the quality and increase the shelf life of beef burgers. Significant antibacterial and antioxidant action has been shown at 2% (w/v) AQP. The findings demonstrated that the treatment of beef burger with AQP decreased the initial total bacterial count and psychrophilic bacteria and extended the shelf-life of beef burger in comparison to the control (beef burger with no addition of AQP, butylated hydroxytoluene (BHT) or gamma radiation treatment). On the other hand, there was no substantial difference in the overall amount of mold and yeast or coliform at zero time. According to sensory characteristics, beef burgers had a shelf life of 6 days for controls and 9, 12, and 15 days for AQP-treated samples at 0.5, 1 and 2%, respectively, compared to γ- irradiated samples, 9 and 21 days, at 3 and 5 Kilo Gray (KGy), respectively.

**Conclusions:**

This research provides a natural red pigment from *Talaromyces purpureogenus* with potent biological activities as antimicrobials and antioxidants to be applied as coloring, additive, and preservative agent in the food industry. Also, the tested pigment offers a powerful alternative to gamma irradiation for extending the shelf life of food products.

## Background

Many synthetic food colorants have been banned due to potential negative effects including hyperactivity in children, allergenicity, toxicological, and carcinogenicity issues. Therefore, researchers have become increasingly interested in accessing naturally colored preservative foods to avoid the danger of the current artificial food additives, which pose a major risk to public health, leading consumers to constantly seek fresh, healthy, safe foods free of any artificial additives [1]. Pigments are coloring compounds having important properties that are significant to numerous industries. They are utilized as additives, antioxidant, color intensifiers, and other functions in food industry. Companies have chosen to use natural color that derived from plant or animal sources. These additives, however, frequently aren't available all year round and have a number of disadvantages, such as instability and low water solubility [2, 3]. Red coolers other than those derived from plants, which cannot be used across the entire pH range, are desperately needed. Currently, the majority of red food coloring comes from insects (carmine), which is one of the most heat- and light-resistant natural red food colorings. However, carmine raises ethical concerns for some social groups. Additionally, carmine goods have the following disadvantages: (i) they are expensive; (ii) they are allergic; and (iii) they are not suited for vegans, vegetarians, koshers, or Halal [4].

As a result, the academic community and the business world are becoming more and more interested in the widely available natural sources of red pigments. Fungi are well known among non-traditional sources for producing an incredible variety of pigments that are frequently more stable and soluble than plant pigments. It is possible for filamentous fungi to produce a wide range of pigment colors, including yellow, orange, red, brown, chestnut, and bronze [5]. Recent studies have shown conclusively that fungi-derived pigments are preferable to synthetic and plant-derived pigments in terms of stability, availability due to lack of seasonal changes, cost-effectiveness, high yield through strain improvement, and easy extraction through downstream processing [6]. Additionally, some of these synthetic dyes are dangerous to use, can cause cancer, irritate the skin and eyes, and are not biodegradable; as a result, they accumulate on land and in rivers, posing ecological issues [7].

Fungi have a crucial hand in the development of pigment with a high yield among the many microorganisms [8]. Numerous primary metabolites, which fungi make and need for their own metabolism, as well as secondary metabolites are produced (not required for its own maintenance). When essential nutrients are lacking or the environment is hostile, secondary metabolites known as fungal pigments are occasionally produced [9]. Many bio-colors are produced by different fungal strains such as *Aspergillus, Fusarium, Penicillum*, and *Trichoderma* [10]. There are too many filamentous fungi that can create an endless variety of colors and are therefore widely available and sustainable. The enormous variety of colors that filamentous fungus can make includes flavins, melanins, azaphilones, anthraquinones, phenazines, carotenoids, quinones, violacein, and indigo [11]. The most common kind of pigment that has been shown to be potentially safe is anthraquinone [12]. They are a group of natural chemicals that have a wide range of biological functions and potential applications. Plants and microorganisms among living organisms produce the majority of them. Anthraquinones and their analogues exhibit a variety of biological activities, including antimicrobial, antiviral, anticancer, enzyme inhibitory, immune stimulant, antiplatelet aggregation and anti-plasmodium activity [13].

The growth of microorganisms and lipid oxidation are the primary factors in food quality degradation and shorter shelf life. Chemical additives are widely used in food products to reduce lipid oxidation, stop the growth of microorganisms, and extend shelf life. Recent investigations, however, indicate that these synthetic substances may be connected to a number of health hazards, including cancer and carcinogenesis which would negate any positive benefits for the user. Food experts are increasingly replacing these synthetic antioxidants with natural ones, which are supposedly safer, due to these safety concerns [14–16].

By giving hydrogen or electrons to free radicals (R**•**, ROO•, and RO•) that are in the process of oxidizing. Primary antioxidants, such as butylated hydroxytoluene (BHT), halt the process and create more stable products. Rather than halting the radical chain reaction, secondary antioxidants delay and slow down the rate of oxidation in order to prevent the oxidation of lipids. The term "multiple-function antioxidants" refers to some antioxidants that may display more than one mechanism [17].

Synthetic antioxidants like butylated hydroxyanisole (BHA), butylated hydroxytoluene (BHT), and tert-butyl hydroquinone (TBHQ) are frequently employed in the food industry as food additives and potential inhibitors of lipid oxidation to address the stability issues of oils and fats [18].

Irradiation has become a productive technology that improves the microbial safety of food. And up to this point, it has been regarded as the most thorough non-thermal food disinfection technique [19]. The irradiation will result in physical chemical changes in the cells. The formation of the cross-ties intra-molecularly follows the loss of nitrogen bases, termination of hydrogen bonding, termination of the sugar phosphate chains from each of the DNA polinukleotida (single strand break), and termination of the adjacent chains on both polinukleotida (double strand break) (base damage) [20].

The target of the present investigation was to study the possibility of using anthraquinone pigment (AQP) as a natural preservative compared to gamma irradiation to prolong the shelf life of the beef burger and improve its quality hygienic by determining the total volatile basic nitrogen (TVBN) and thiobarbituric acid reactive substances (TBARS) and their microbiological content in fresh samples and stored for 24 days.

## Results

### Pigment production and extraction

In a prior study [21], vital parameters such as fermentation media, temperature, pH, and incubation time influenced pigment production, and their optimum levels were found by researchers using the Box-Behnken Design and Response Surface approach (BBD and RSM) to obtain a high pigment output. The numerical optimization method yielded the following optimal conditions: In YMB medium, pH 6 was achieved at a temperature of 25 °C for 18 days.

Produced pigment was extracted using various solvents to determine a suitable one for extraction. According to our observations (Table 1), pigment was insoluble in nonpolar solvents. Ethyl acetate is partially soluble, but butanol and ethanol are completely soluble. As a result, the antibacterial activity of soluble extracted pigment was investigated in search of the most effective extraction solvent. Ethanol extracts had remarkable antibacterial activity against all pathogenic microorganisms tested, as shown by the measurement of the zone of inhibition. A maximum inhibition zone of 5.1 ± 0.10 cm in ethanolic extract was recorded against Gram-positive *staphylococcus aureus*. In the instance of the Gram-negative bacteria, the maximum zone of inhibition was 4.60 ± 0.10 cm for *Pseudomonas aeruginosa* in ethanol. Also, pigment dissolved in butanol displays a maximal inhibitory zone against *Pseudomonas aeruginosa* and *staphylococcus aureus* (4.13 ± 0.35 and 4.13 ± 0.15 cm respectively). The results also showed that maximum inhibition zones in ethyl acetate were 4.80 ± 0.20 cm for *Bacillus cereus*. Table 2 displays the antibacterial activities of soluble pigment in various solvents against Gram-positive and Gram-negative microorganisms. It is worth noting that none of the tested solvents have any activity against pathogenic bacteria at the tested volume of 20 µl [22].

**Table 1:** Absorbance of extracted pigment under extraction with different solvents

Solvent		Polarity index	Solubility of pigment	Absorbance (510 nm)	
				before extraction	after extraction
Diethyl ether	Non polar	2.8	insoluble	2.1	0.0
Chloroform		4.1	insoluble	2.1	0.0
Hexane		0.1	insoluble	2.1	0.0
n-Butanol	Polar	3.9	Complete soluble	2.1	1.9
Ethanol		0.6	Complet soluble	2.1	2.0
Ethyl acetate		4.4	Moderate soluble	2.1	1.7

**Table 2:** Antibacterial activity of extracted pigment with different solvents

Bacteria	Organic solvents		
	Ethanol	n-Butanol	Ethyl acetate
	(Inhibition zone, cm ± SD)		
Gram –ve			
* Escherichia coli*	4.27 ± 0.57	3.47 ± 0.55	3.67 ± 0.42
* Salmonella typhimurium*	4.30 ± 0.20	3.83 ± 0.35	3.90 ± 0.30
* Klebsiella pneumonia*	3.30 ± 0.20	2.60 ± 0.10	2.60 ± 0.10
* Pseudomonas aeruginosa*	4.60 ± 0.10	4.13 ± 0.35	4.30 ± 0.20
Gram + ve			
* Bacillus cereus*	4.17 ± 0.15	3.7 ± 0.20	4.80 ± 0.20
* Bacillus subtilis*	4.40 ± 0.17	2.80 ± 0.65	3.40 ± 0.21
* Staphylococcus aureus*	5.1 ± 0.10	4.13 ± 0.15	3.93 ± 0.15
* Listeria inanovii*	4.60 ± 0.10	4.00 ± 0.10	3.9 ± 0.10

*SD* Standard Deviation

### LC mass spectroscopy

**Fig. 1:**
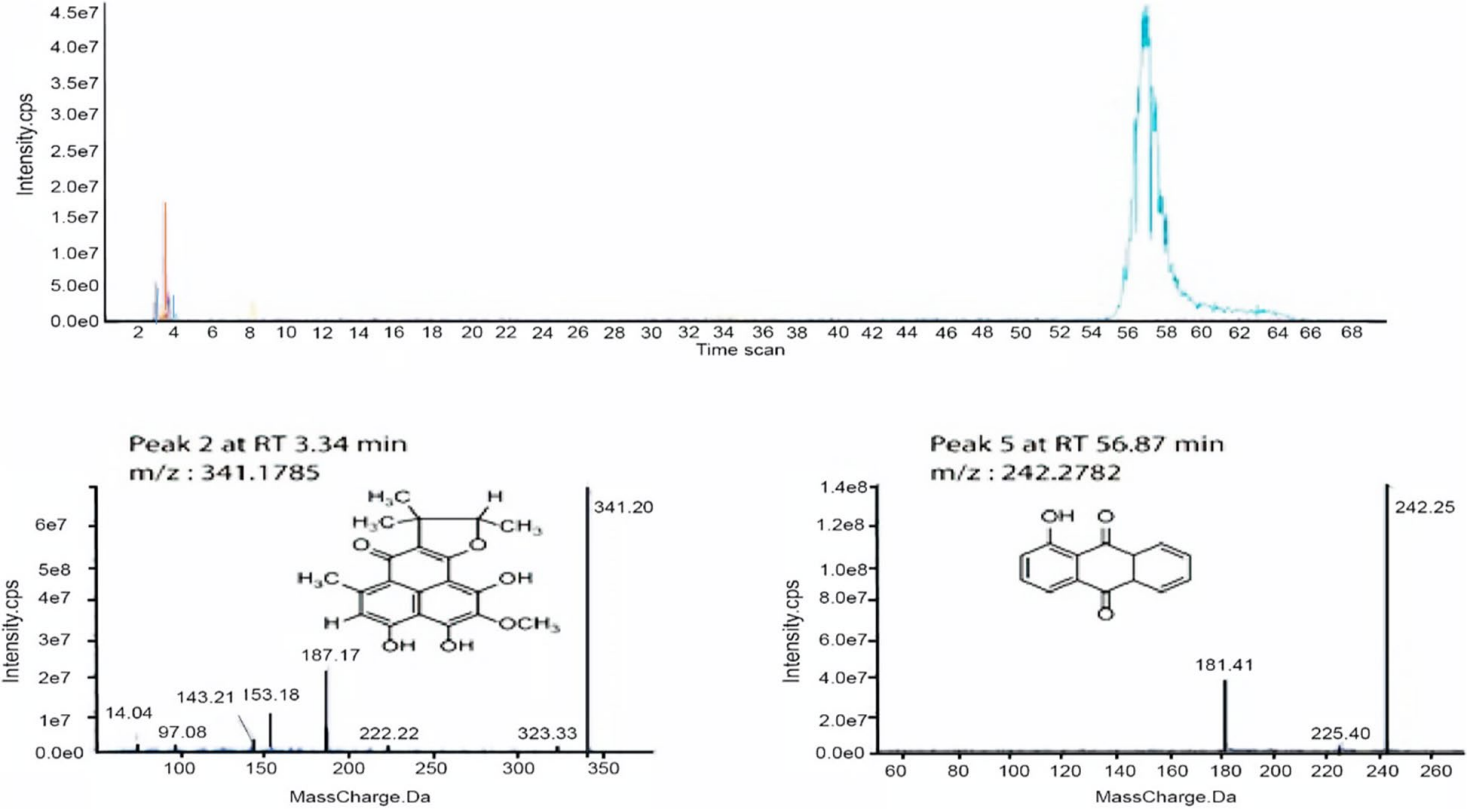
Base peak chromatogram of *penicillium propgonum* pigments extract and identified secondary metabolites which are: Herqueinone (2), Anthraquinone (5). Meas m/z implies measured m/z

The LC spectrum detects five different peaks at a wavelength of 275 mAU for *Talaromyces purpureogenus* extract, among them two major peaks was severely detected. As shown in Fig. 1, the mass value of the dominant peak was 242 followed by 342 Daltons *m/z*, the chromatographic spectrum was ranged between 175– 342 Daltons *m/z*. Those mass values were found to match specific certain compounds isolated previously. The mass spectroscopy of *Talaromyces purpureogenus* extract is shown in Fig. 1.

A mass screening on the above spectrum was conducted and summarized as follows, the screened area from 2.85–56.87 min showed specific mass value peaks, two major peaks were detected and studied and analyzed extensively. Detected mass values were corresponded to previously identified compounds. The major red pigments isolated from the crude extract belongs to anthraquinone chemical compounds including, anthraquinone as dominant compound, followed by herqueinone as showed in Fig. 1.

### Fourier transform infrared spectroscopy FTIR

**Fig. 2:**
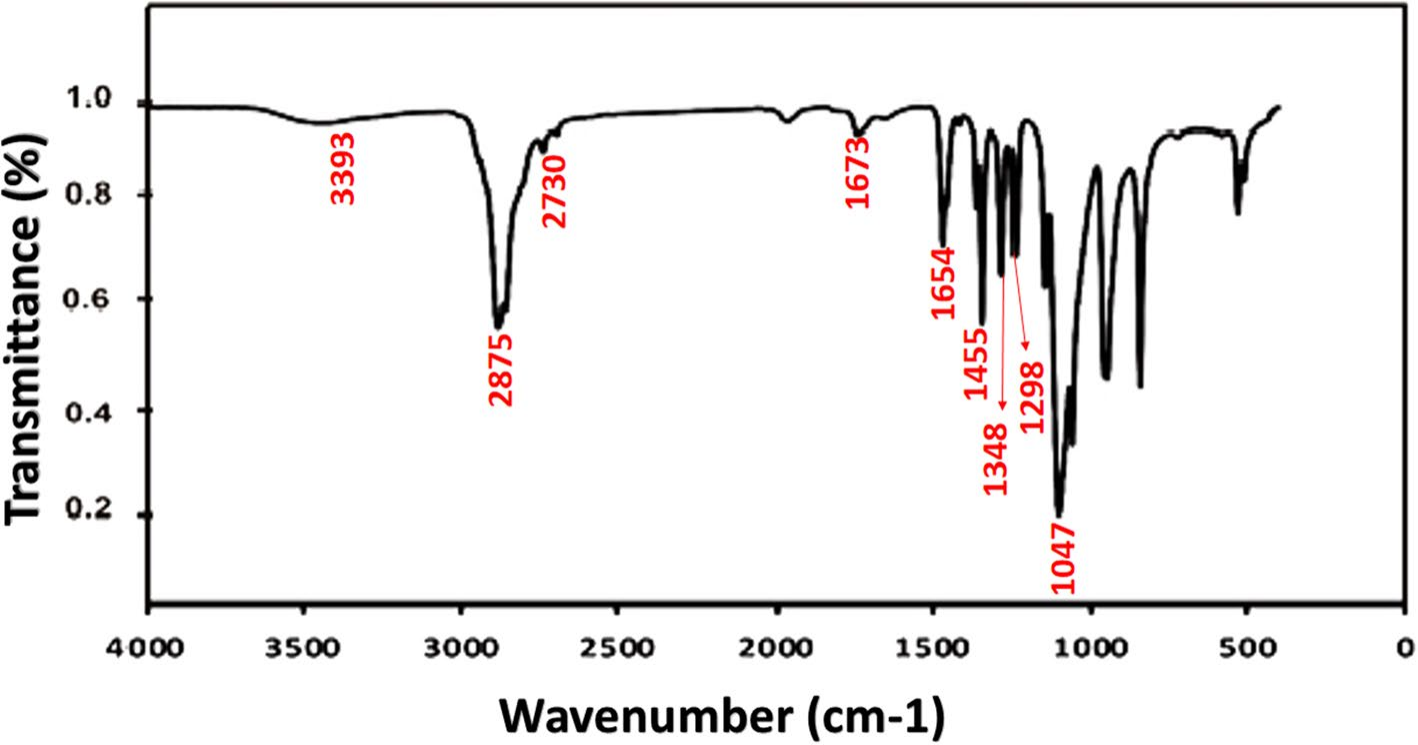
Fourier-transform infrared spectroscopy (FTIR) spectra crude pigment extract

FTIR is a technique used in structure identification studies, where the resulted FTIR spectrum of the crude extract is expressing the integral functional groups in the compounds. FTIR spectra of crude pigment extract are presented in Fig. 2, with the following absorptions bands: The absorption band at 3393 cm^−1^corresponding to hydroxyl group O–H stretching vibration. The band at 2875 cm^−1^ is due to alkane C–H stretching vibration where the band at 2730 is weak, 1-OH. The FTIR spectrum peak of carbonyl C = O stretch appeared at 1673 and 1645 cm^−1^. The bands at 1455–1348 cm^−1^ are attributed to aromatic C = C stretch, and the aromatic C–O stretches at 1298 and 1047 cm^−1^. The obtained data supports the reported FTIR spectrum of anthraquinones [23, 24].

### Effect of gamma irradiation on antimicrobial activity of pigment

Because gamma rays are used to preserve and extend the shelf life of foods, it was vital to investigate the influence of irradiation on the food pigment's antibacterial characteristics. Table 3 shows the effects of exposing the AQP to gamma radiation at various doses of 1, 3, 5 kGy. The findings revealed that irradiation using gamma rays had no significant effect on the antimicrobial capabilities of AQP. Radiation had a slight effect against all tested microorganisms, oscillating between a decrease and an increase. As a result of the findings, the study concluded that there is no reason to avoid using irradiation in the case of foods containing the AQP.

**Table 3:** Antimicrobial activity of irradiated anthraquinone pigment

Micoorganisms	Pigment irradiated with different Gamma rays dose (KGy)			
	**0**	**1**	**3**	**5**
	**(Inhibition zone, cm ± SD)**			
**Gram –ve bacteria**				
*** Escherichia coli***	4.33 ± 0.15	4.10 ± 0.20	4.10 ± 0.17	4.00 ± 0.17
*** Salmonella typhimurium***	4.67 ± 0.35	4.27 ± 0.32	4.83 ± 0.15	3.93 ± 0.25
*** Klebsiella pneumonia***	3.47 ± 0.12	3.50 ± 0.26	3.07 ± 0.15	3.30 ± 0.26
*** Pseudomonas aeruginosa***	4.50 ± 0.20	4.27 ± 0.25	4.10 ± 0.30	4.23 ± 0.31
**Gram + ve bacteria**				
*** Bacillus cereus***	4.00 ± 0.10	4.03 ± 0.06	3.87 ± 0.50	3.77 ± 0.21
*** Bacillus subtilis***	4.33 ± 0.21	4.03 ± 0.25	4.03 ± 0.15	4.07 ± 0.21
*** Staphylococcus aureus***	4.00 ± 0.26	4.03 ± 0.15	3.90 ± 0.10	4.03 ± 0.15
*** Listeria inanovii***	4.90 ± 0.26	4.83 ± 0.25	4.77 ± 0.25	4.57 ± 0.25
**Fungi**				
*** Aspergillus niger***	N.I	N.I	N.I	N.I
*** Fusarium oxysporum***	1.43 ± 0.15	1.24 ± 0.25	1.16 ± 0.30	N.I
*** Alternaria alternata***	1.27 ± 0.12	1.20 ± 0.32	1.17 ± 0.10	1.22 ± 0.34
*** Alternaria solani***	N.I	N.I	N.I	N.I
**yeast**				
*** Candida albicans***	1.60 ± 0.10	1.9 ± 0.20	1.27 ± 0.25	N.I

***N.I*** No Inhibition

### Antioxidants activity on DPPH and ABTS

The antioxidant activity was determined by the potential scavenging of two artificial radical DPPH and ABTS. In this assay, we tested the antioxidants potential of non irradiated pigments crude extract and that treated with gamma rays at three different doses (1, 3, and 5 KGy). In DPPH assay, the crude pigment extract displayed the highest antioxidants potencies compared to that obtained by the irradiated crude pigment (Table 4). However, it was clearly observed that the antioxidants activity has significantly reduced by increasing γ-ray dose. Indicating that, gamma rays might inhibits the antioxidants activity of the crude dye. The same patterns were observed in ABTS assay with slightly increasing in the antioxidants activity (Table 4). The concentration that induces 50% of activity (IC_50_) was calculated from the plotting % Inhibition against concentration (data not showed). In general, the findings revealed that irradiation has a minor detrimental or favorable influence on antimicrobial properties. However, it's worth mentioning that irradiation has a considerable negative impact on the AQP's antioxidant activity, so it shouldn't be used to preserve foods containing anthraquinone pigment.

**Table 4:** Antioxidant activity of crude pigment extract and γ-ray treated samples using DPPH and ABTS assay

DPPH assay				
**Conc. (µg.ml**^**−1**^**)**	**Crude pigment**	**1 kGy**	**3 kGy**	**5 kGy**
0.05	21.8 ± 2.44	15.52 ± 0.65	10.96 ± 4.38	1.16 ± 1.14
0.1	31.86 ± 3.07	20.58 ± 0.62	15.98 ± 0.51	3.45 ± 1.02
0.15	45.94 ± 0.44	28.23 ± 0.88	23.05 ± 1.12	7.04 ± 0.51
0.2	55.18 ± 1.54	42.22 ± 2.81	27.68 ± 0.71	12.06 ± 2.25
0.5	65.36 ± 3.88	61.06 ± 2.02	32.93 ± 1.90	16.20 ± 0.61
1	84.13 ± 4.08	75.13 ± 1.36	42.56 ± 0.68	20.03 ± 1.03
**Conc. (µg.ml**^**−1**^**)**	**ABTS assay**			
	**Crude pigment**	**1 kGy**	**3 kGy**	**5 kGy**
0.05	19.68 ± 0.02	19.13 ± 2.18	15.14 ± 1.84	0.99 ± 0.78
0.1	40.54 ± 0.03	29.70 ± 0.69	22.44 ± 0.27	3.09 ± 1.09
0.15	56.50 ± 0.01	38.4 ± 0.51	30.52 ± 1.17	5.17 ± 0.54
0.2	66.30 ± 0.72	49.47 ± 0.64	45.62 ± 1.52	9.38 ± 1.60
0.5	75.73 ± 0.07	66.93 ± 0.75	53.42 ± 3.45	15.64 ± 1.38
1	87.75 ± 0.01	77.86 ± 0.82	69.79 ± 1.56	18.62 ± 0.5

### DNA damage protection

**Fig. 3:**
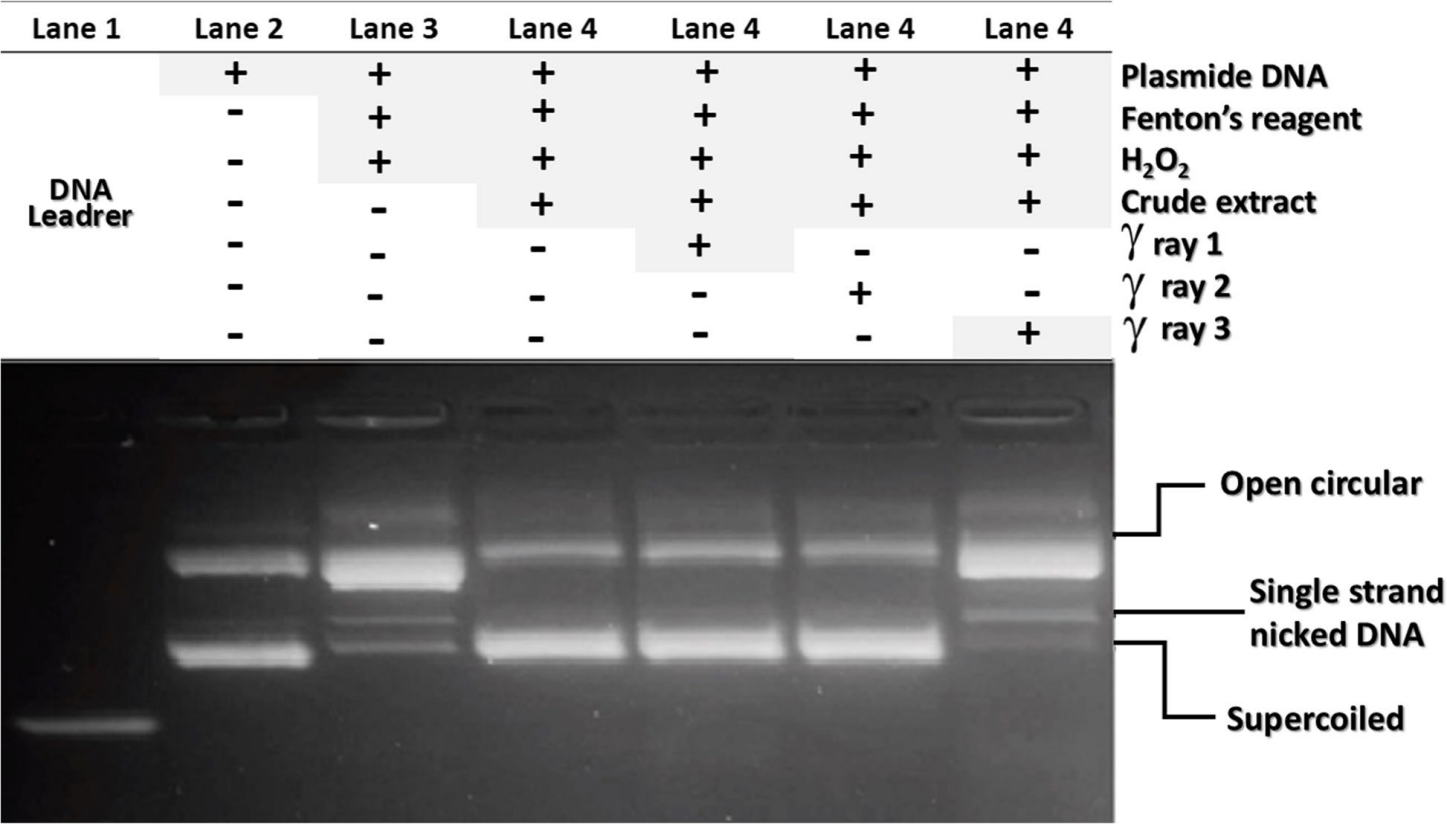
In vitro analysis of DNA damage protection control. Lane 1: DNA ladder, Lane 2: RHN1 DNA Plasmid, Lane 3: RHN1 DNA Plasmid treated with Fenton's reagent, Lane 4: crude extract, Lane 5: X-ray1, Lane 6: Gamma rays2 and Lane 7: Gamma rays3 at 0.5 µg/ml, respectively. All the reaction mixtures were incubated for 20 min at 37 °C

Oxidative stress induced by Fenton's reagent generates OH radical that causes DNA damage and therefore, disrupt the plasmid DNA forms and density separated by agarose gel. Thus we tested the potential protection effects of AQP on the DNA damage treated with Fenton's reagent compared to respective controls. After incubation with crude extract exposed or not to γ-ray, *RHN1* plasmid DNA was separated on agarose gel electrophoresis. In general, from two to three distinguished forms of Plasmid DNA are detected, The open circular appear first followed by linearized DNA form and the supercoiled circular DNA appear as last thick band. However, oxidative stress by Fenton reaction induces DNA single or double-stranded breaks, thus an alteration in the bands density are formed [25]. As a result, *RHN1* supercoiled circular form was slightly degraded and the linear band was observed in positive control treated with Fenton's reagents alone (lane 3). The crude pigment extract showed high potential DNA damage protection compared to crude pigments exposed or not with gamma rays dose 1 kGy and dose 3 kGy displayed potential DNA damage protection (lanes 4–6, respectively Fig. 3), indicating that crude pigments gains the scavenging activity against OH^•^ radical generated by Fenton's reagents and protects DNA from damage in response to OH^•^ radical compared to respective control. However, no significant difference was observed with crude pigments treated with γ-ray at dose of 3 KGy (lanes 7). Altogether, the results indicated that crude dye enhances the protection capacity of DNA damage induced by Fenton's reagent compared to untreated control. In addition, the high dose of γ-ray may alter the antioxidants components and decrease the radical scavenging activity of the crude extract. However, the previous data was in agreement with the antioxidants obtained data.

### Cytotoxic activity

In order to test the cytotoxic effects of AQP and gamma raystreated pigment, two normal cell lines were used in this experiment. The results indicate that; no significant cytotoxic effects were observed against tested normal cell using crude extract. The results showed that crude AQP extract has no cytotoxic effects against MCF12F and Bj-1 normal cell lines with IC_50_ values 490 and 578 µg/ml, respectively (Table 5). Indicating that crude AQP extract could be used successfully and safely used as food supplement. However, the crude extract treated with gamma rays showed a high cytotoxic activity relative to high gamma rays dose. The IC_50_ values against MCF12F were 87, 57 and 23 µg.ml^−1^ at gamma rays dose 1, 2 and 3 KGy, respectively. While, The IC_50_ values against BJ-1 were 94, 72 and 17 µg.ml^−1^, respectively. These results indicated that using gamma rays treated pigments increased the cytotoxic effects of the crude pigments extract against Bj-1 and MCF12F normal cell lines.

**Table 5:** Cytotoxic activity of crude pigment extract, γ-ray treated samples against normal cells

Cell line	Cytotoxic IC_50_ (µg.ml^−1^)			
	**Crude pigment**	**1 kGy**	**3 kGy**	**5 kGy**
**MCF12F**	490.46 ± 7.12	87.14 ± 1.67	57.34 ± 0.67	23.15 ± 1.62
**BJ-1**	578.03 ± 5.76	94.56 ± 3.34	72.55 ± 2.49	17.30 ± 1.51

## Effect of AQP, BHT, and -irradiation on beef burger microbial characteristics during cold storage (4 ± 1 °C)

### Total bacterial count (cfu/g)

According to Table 6's findings, all beef burger treatments had total bacterial counts (TBC) that ranged from 3.3 × 10^3^ to 5.7 × 10^5^ at zero time, with the control sample having higher counts (5.7 log cfu/g) than the other treatments. Gamma irradiation resulted in the lowest initial overall bacterial count (5 kGy). These findings concur with those provided by Fallah [26]. The TBC count increased steadily over the course of cold storage in both the control and treatment samples compared to their baseline counts with longer storage times, though more quickly in the control sample. After 9 days of cold storage (4 ± 1 °C), the TBC count reached 4.1 × 10^7^ cfu/g. Additionally, the shelf-life of burger samples was extended to 9, 12, 15, 9, and 21 days, respectively, compared to 6 days in the control sample, by synthesising the samples with AQP (0.5, 1 and 2 percent), BHT, and irradiation treatment at dosage levels of 3 and 5 kGy. After 12, 15, 18, 12, 12 and 24 days of cold storage, the TBC reached 3.5 × 10^7^, 2.6 × 10^7^, 1.7 × 10^7^, 3.7 × 10^7^, and 2.3 × 10^7^ and 3.7 × 10^7^ cfu/g, respectively, at the end of the storage period.

**Table 6:** Total bacterial counts (cfu.g^−1^) of beef burger affected by anthraquinone pigment (AQP), BHT and γ- irradiation during cold storage (4 ± 1ºC)

Storage Period (day)	Treatments						
	**control**	**Anthraquinone concentration (%)**			**BHT (200 ppm)**	**γ- irradiation (kGy)**	
		**0.5**	**1.0**	**2.0**		**3**	**5**
**0**	**5.7 × 10**^**5**^	**5.6 × 10**^**5**^	**5.5 × 10**^**5**^	**5.2 × 10**^**5**^	**5.2 × 10**^**5**^	**4.8 × 10**^**4**^	**3.3 × 10**^**3**^
**3**	**3.3 × 10**^**6**^	**9.6 × 10**^**5**^	**8.3 × 10**^**5**^	**6.1 × 10**^**5**^	**7.7 × 10**^**5**^	**8.3 × 10**^**4**^	**4.5 × 10**^**3**^
**6**	**8.3 × 10**^**6**^	**3.7 × 10**^**6**^	**1.1 × 10**^**6**^	**8.8 × 10**^**5**^	**3.5 × 10**^**6**^	**2.6 × 10**^**5**^	**8.6 × 10**^**3**^
**9**	**4.1 × 10**^**7**^ **R**	**8.4 × 10**^**6**^	**4.3 × 10**^**6**^	**2.5 × 10**^**6**^	**7.7 × 10**^**6**^	**5.7 × 10**^**6**^	**2.9 × 10**^**4**^
**12**		**3.5 × 10**^**7**^ **R**	**7.2 × 10**^**6**^	**4.4 × 10**^**6**^	**3.7 × 10**^**7**^ **R**	**2.3 × 10**^**7**^ **R**	**7.5 × 10**^**4**^
**15**			**2.6 × 10**^**7**^ **R**	**7.2 × 10**^**6**^			**3.4 × 10**^**5**^
**18**				**1.7 × 10**^**7**^ **R**			**1.6 × 10**^**6**^
**21**							**8.8 × 10**^**6**^
**24**							**3.7 × 10**^**7**^ **R**

*R* rejected

### Psychrophilic bacteria (cfu/g) in beef burgers stored at a low temperature (4 ± 1 °C)

According to data in Table 7, the control sample's initial psychrophilic bacterial count (PBC) was 4.5 × 10^4^ cfu/g. The addition of AQP (0.5, 01, and 02 percent) and BHT had no appreciable impact on the samples' initial PBCs, which were 4.3 × 10^4^, 4.4 × 10^4^, 3.9 × 10^4^, and 4.3 × 10^4^ cfu/g, respectively. In addition, compared to the control sample and other treatments at zero time, the irradiation treatment at 3 and 5 kGy had decreased the initial count to 2.2 × 103 and 1.9 × 10^2^ cfu/g, respectively. However, total PBC counts gradually increased for all burger samples stored at 4 1 °C because to higher rates in the control samples, where its count peaked at 5.7 × 106 cfu/g at the time of rejection after 9 days. PBC counts decreased with the addition of AQP by 0, 1, and 2 percent, reaching 4.8 × 10^6^, 2.2 × 10^6^, and 9.2 × 10^5^ cfu/g after 12, 15, and 18 days of cold storage, respectively, as opposed to 5.5 × 10^6^ and 2.1 × 105 cfu/g in the samples containing BHT and GI3 after 12 days. In contrast to all other treatments, the irradiated samples at 5 kGy showed the lowest rate of rise during the duration of cold storage, with a PBC of 6.6 × 10^5^ cfu/g after 24 days of storage.

**Table 7:** Total psychrophilic bacteria (cfu.g^−1^) of beef burger affected by anthraquinon pigment (AQP), BHT and γ- irradiation during cold storage (4 ± 1ºC)

Storage Period (day)	Treatments						
	**control**	**Anthraquinone concentration (%)**			**BHT (200 ppm)**	**γ- irradiation (kGy)**	
		**0.5**	**1.0**	**2.0**		**3**	**5**
**0**	**4.5 × 10**^**4**^	**4.3 × 10**^**4**^	**4.4 × 10**^**4**^	**3.9 × 10**^**4**^	**4.3 × 10**^**4**^	**2.2 × 10**^**3**^	**1.9 × 10**^**2**^
**3**	**2.8 × 10**^**5**^	**8.1 × 10**^**4**^	**6.1 × 10**^**4**^	**4.9 × 10**^**4**^	**9.0 × 10**^**4**^	**5.5 × 10**^**2**^	**4.7 × 10**^**2**^
**6**	**8.1 × 10**^**5**^	**2.7 × 10**^**5**^	**1.3 × 10**^**5**^	**8.2 × 10**^**4**^	**4.1 × 10**^**5**^	**2.5 × 10**^**3**^	**9.3 × 10**^**2**^
**9**	**5.7 × 10**^**6**^ **R**	**8.6 × 10**^**5**^	**4.1 × 10**^**5**^	**2.5 × 10**^**5**^	**9.2 × 10**^**5**^	**3.4 × 10**^**4**^	**2.5 × 10**^**3**^
**12**		**4.8 × 10**^**6**^ **R**	**8.3 × 10**^**5**^	**5.1 × 10**^**5**^	**5.5 × 10**^**6**^ **R**	**2.1 × 10**^**5**^ **R**	**7.9 × 10**^**3**^
**15**			**2.2 × 10**^**6**^ **R**	**6.8 × 10**^**5**^			**3.4 × 10**^**4**^
**18**				**9.2 × 10**^**5**^ **R**			**7.7 × 10**^**4**^
**21**							**1.5 × 10**^**5**^
**24**							**6.6 × 10**^**5**^** R**

*R* rejected

### Coliform group bacteria (cfu/g) in beef burgers stored at a low temperature (4 ± 1 °C)

The samples incorporated with AQP (0.5, 1 and 2 percent) and BHT had no appreciable changes in the counts compared to the control sample, where their counts were 2.6 × 10^2^, 2.5 × 10^2^, 2.3 × 10^2^, and 2.8 × 10^2^ cfu/g, respectively. Table 8 showed that the initial coliform group counts (CGC) were 2.7 × 10^2^ cfu/g in control samples. When compared to other treatments, the treatment of GI3 and GI5 had completely removed CGC in beef burger samples for the entire term of cold storage. The control sample among the experimental groups showed the fastest growth in CGC (2.0 × 10^3^ cfu/g) after nine days, followed by samples treated with BHT, AQP 0.5 percent, AQP 1 percent, and AQP 2 percent. With the exception of the GI3 and GI 5 treatments, cold storage caused a progressive increase in the CGC for all beef burger samples.

**Table 8:** Coliform group counts (cfu.g^−1^) of beef burger affected by anthraquinone pigment (AQP), BHT and γ- irradiation during cold storage (4 ± 1ºC)

Storage Period (day)	Treatments						
	**control**	**Anthraquinon concentration (%)**			**BHT (200 ppm)**	**γ- irradiation (kGy)**	
		**0.5**	**1.0**	**2.0**		**3**	**5**
**0**	**2.7 × 10**^**2**^	**2.6 × 10**^**2**^	**2.5 × 10**^**2**^	**2.3 × 10**^**2**^	**2.8 × 10**^**2**^	**N.D**	**N.D**
**3**	**4.3 × 10**^**2**^	**4.0 × 10**^**2**^	**4.1 × 10**^**2**^	**3.6 × 10**^**2**^	**3.9 × 10**^**2**^	**N.D**	**N.D**
**6**	**7.2 × 10**^**2**^	**6.9 × 10**^**2**^	**5.4 × 10**^**2**^	**5.2 × 10**^**2**^	**7.1 × 10**^**2**^	**N.D**	**N.D**
**9**	**2.0 × 10**^**3**^ **R**	**9.8 × 10**^**2**^	**7.8 × 10**^**2**^	**6.8 × 10**^**2**^	**1.9 × 10**^**3**^ **R**	**N.D**	**N.D**
**12**		**1.7 × 10**^**3**^ **R**	**9.0 × 10**^**2**^	**8.0 × 10**^**2**^		**N.D** **R**	**N.D**
**15**			**1.6 × 10**^**3**^ **R**	**8.4 × 10**^**2**^			**N.D**
**18**				**1.7 × 10**^**3**^ **R**			**N.D**
**21**							**N.D**
**24**							**N.D** **R**

*R* rejected

### Total yeast and molds count (cfu/g) in beef burgers stored at a low temperature (4 ± 1 °C)

From the data in Table 9, it is clear that the control sample's initial total yeasts and molds count (TYMC) was 4.6 × 10^2^ cfu/g, and that the addition of BHT and AQP at various concentrations of 0.5, 1 and 2 percent had no appreciable impact on the initial count of yeasts and molds at zero time, where their counts were 4.6 × 10^2^, 4.5 × 10^2^, 4.3 × 10^2^, and 4.2 × 10^2^ cfu/g, On the other side, it was seen that TYMC were similarly impacted by the 4 ± 1 °C cold storage duration. The TYMC in all beef burger treatments rose by lengthening the storage duration The control sample saw the largest increase in TYMC over the course of cold storage, followed by samples treated with BHT, AQP 0.5, 1, and 2 percent, whose counts reached 4.2 × 10^4^, 2.9 × 10^4^, 2.6 × 10^4^, 2.3 × 10^4^, 3.9 × 10^4^, and 3.9 × 10^4^ cfu/g after 9, 12, 15, and 18 days of cold storage, respectively. From the data in Table 9, it is clear that the control sample's initial total yeasts and molds count (TYMC) was 4.6 × 10^2^ cfu/g, and that the addition of BHT and AQP at various concentrations of 0.5, 1 and 2 percent had no appreciable impact on the initial count of yeasts and molds at zero time, where their counts were 4.6 × 10^2^, 4.5 × 10^2^, 4.3 × 10^2^, and 4.2 × 10^2^ cfu/g. On the other side, it was seen that TYMC were similarly impacted by the 4 ± 1 °C cold storage duration. The TYMC in all beef burger treatments rose by lengthening the storage duration The control sample saw the largest increase in TYMC over the course of cold storage, followed by samples treated with BHT, AQP 0.5, 1, and 2 percent, whose counts reached 4.2 × 10^4^, 2.9 × 10^4^, 2.6 × 10^4^, 2.3 × 10^4^, 3.9 × 10^4^, and 3.9 × 10^4^ cfu/g after 9, 12, 15, and 18 days of cold storage, respectively. However, the GI3 and GI5 treatments inhibited the growth of yeasts and moulds until 6 and 12 days of cold storage, respectively. After 12 and 24 days of cold storage, the count reached 4.2 × 10 and 5.5 × 10^2^ cfu/g, respectively. These results are very similar to those of Abdeldaiem [27].

**Table 9:** Total yeasts and molds count (cfu.g^−1^) of beef burger affected by anthraquinone pigment (AQP), BHT and γ- irradiation during cold storage (4 ± 1ºC)

Storage Period (day)	Treatments						
	**control**	**Anthraquinone concentration % (W/V)**			**BHT (200 ppm)**	**γ- irradiation (kGy)**	
		**0.5**	**1.0**	**2.0**		**3**	**5**
**0**	**4.6 × 10**^**2**^	**4.5 × 10**^**2**^	**4.3 × 10**^**2**^	**4.2 × 10**^**2**^	**4.6 × 10**^**2**^	**N.D**	**N.D**
**3**	**9.3 × 10**^**2**^	**8.6 × 10**^**2**^	**6.4 × 10**^**2**^	**4.7 × 10**^**2**^	**9.9 × 10**^**2**^	**N.D**	**N.D**
**6**	**7.7 × 10**^**3**^	**4.6 × 10**^**3**^	**8.9 × 10**^**2**^	**6.4 × 10**^**2**^	**6.3 × 10**^**3**^	**N.D**	**N.D**
**9**	**4.2 × 10**^**4**^** R**	**9.8 × 10**^**3**^	**4.6 × 10**^**3**^	**1.5 × 10**^**3**^	**1.3 × 10**^**4**^	**1.3 × 10**	**N.D**
**12**		**2.9 × 10**^**4**^ **R**	**7.9 × 10**^**3**^	**5.8 × 10**^**3**^	**3.9 × 10**^**4**^ **R**	**4.2 × 10** **R**	**N.D**
**15**			**2.6 × 10**^**4**^ **R**	**7.8 × 10**^**3**^			**1.3 × 10**
**18**				**2.3 × 10**^**4**^ **R**			**3.3 × 10**
**21**							**8.4 × 10**
**24**							**5.5 × 10**^**2**^ **R**

*R* rejected

## Effect of AQP, BHT and γ-irradiation on the physicochemical characteristics of beef burger during cold storage (4 ± ˚C)

### TVBN (mg N/100 g) on wet weight bases of beef burger during cold storage (4 ± 1ºC)

**Fig. 4:**
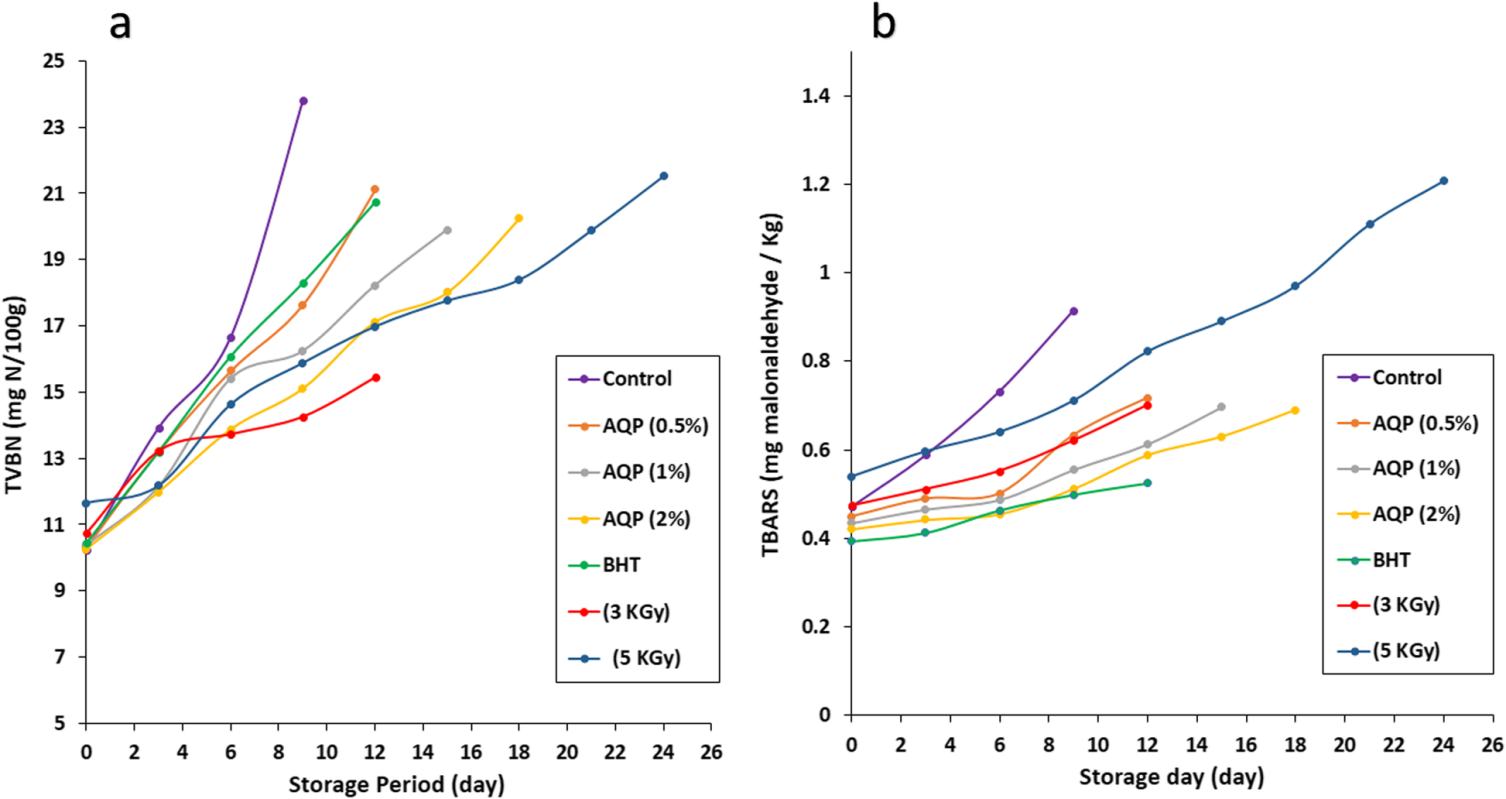
**a** Total volatile basic nitrogen of beef burger affected by anthraquinon pigment (AQP), BHT and γ- irradiation during cold storage (4 ± 1ºC) (mg N/ 100gm). **b** Thiobarbituric acid reactive substances of beef burger affected by anthraquinon pigment (AQP), BHT and γ- irradiation during cold storage (4 ± 1ºC) (mg /100 g sample)

All beef burger samples were compared to a control sample to examine the effect of addition AQP, BHT, and gamma irradiation on the total volatile basic nitrogen (TVBN). The results are shown in Fig. 4a. It is evident that, when compared to control samples at 0 time, there were no appreciable variations in the TVBN concentration between the samples containing AQP (0.5, 1 and 2 percent) and BHT. Furthermore, compared to control samples and other treatments, the TVBN content of the irradiated (3 and 5 kGy) samples was considerably greater (p 0.05). The observed results showed that, depending on the examined treatment, the TVBN values for all of the burger samples gradually and significantly rose as storage time increased. After 9 days of cold storage, the control sample exhibited the highest TVBN concentration (23.8 ± 0.1 mg/100 g sample). While in storage, the various beef burger samples with AQP (0.5, 1 and 2 percent), BHT, GI3 and GI5 showed lower TVBN content than the control sample, with values of 21.11 ± 0.01, 19.9 ± 0.02, 20.25 ± 0.017, 20.75 ± 0.026, 15.45 ± 0.105 and 21.52 ± 0.03 mg/100 g sample after 12, 15, 18, 12, 12 and 24 days, respectively.

### TBARS (mg malonaldehyde /kg on wet weight bases) of beef burger during cold storage (4 ± 1ºC)

Malonaldehyde (MDA), a subsequent product of lipid oxidation that may give oxidised fat an unpleasant flavor, is measured by TBARS analysis. The initial values for all treatments were reached to 0.449 ± 0.002, 0.434 ± 0.005, 0.421 ± 0.002, 0.393 ± 0.002, 0.474 ± 0.003 and 0.539 ± 0.001 mg MDA/kg for AQP (0.5, 1 and 2 percent), BHT, and -irradiation (3 and 5 kGy), respectively, as opposed to 0.472 ± 0.001 mg MDA/kg in control samples. These results are shown in Fig. 4b. The findings demonstrated a substantial difference between the BHT-containing and irradiated burger samples and the control sample. While the sample's TBARS values increased after receiving the 5 kGy of radiation treatment. At the end of the storage period after 9, 12, 15, 18, 21, and 24 days for control, AQP (0.5, 1 and 2 percent), BHT, GI3 and GI5, respectively, it is evident that the cold storage at 4 °C significantly increased the TBARS gradually for all samples (Fig. 4b).

### Sensory characteristics of beef burger samples as influenced by various AQP, BHT, and gamma irradiation levels during cold storage at 4 ± 1 °C

Tables 10, 11 and 12 list the sensory characteristics of beef burgers as they are impacted by AQP concentrations of 0.5, 1, and 2 percent in comparison to the control, as well as gamma irradiation at dose levels of 3 and 5 kGy during cold storage at (4 1 °C) for 24 days.

**Table 10:** Appearance of beef burger affected by anthraquinone pigment (AQP) and γ- irradiation during cold storage (4 ± 1ºC)

Storage Period (day)	Treatments						
	**control**	**Anthraquinon concentration (%)**			**BHT (200 ppm)**	**γ- irradiation (kGy)**	
		**0.5**	**1.0**	**2.0**		**3**	**5**
**0**	**9.30**^Aa^ ** ± **0.483	**9.20**^Aab^ ** ± **0.422	**9.18** ^Aab^ ** ± **0.336	**8.83**^Ab^ ** ± **0.585	**9.25** ^Aab^ ** ± **0.425	**9.10** ^Aab^ ** ± **0.568	**8.90** ^Aab^ ** ± **0.316
**3**	**8.51**^Ba^ ** ± **0.517	**8.61** ^Ba^ ** ± **0.458	**8.82** ^Aa^ ** ± **0.382	**8.73** ^Aa^ ** ± **0.442	**8.75** ^Aa^ ** ± **0.717	**8.70** ^ABa^ ** ± **0.675	**8.70** ^Aa^ ** ± **0.483
**6**	**6.74** ^Cc^ ** ± **0.795	**7.44** ^Cb^ ** ± **0.685	**7.56** ^Bb^ ** ± **0.685	**8.21**^Bb^ ** ± **0.612	**7.35** ^Bf^ ** ± **0.474	**8.20** ^BCa^ ** ± **0.632	**8.77**^Aa^ ** ± **0.416
**9**	**3.52**^De^ ** ± **0.812	**5.17**^Dd^ ** ± **0.596	**6.64**^Cc^ ** ± **0.479	**7.81**^Cb^ ** ± **0.910	**5.58**^Cd^ ** ± **0.689	**7.70**^Db^ ** ± **0.675	**8.54** ^Aa^ ** ± **0.499
**12**		**2.82**^Ecd^ ** ± **0.382	**5.33**^Db^ ** ± **0.667	**7.13**^ Da^ ** ± **0.693	**3.05**^Dc^ ** ± **0.599	**2.40**^Ed^ ** ± **0.699	**7.63**^Ba^ ** ± **0.914
**15**			**3.41**^Ec^ ** ± **0.509	**5.62**^Eb^ ** ± **0.683			**6.96**^Ca^ ** ± **0.951
**18**				**3.21**^Fb^ ** ± **0.612			**6.28**^ Da^ ** ± **0.934
**21**							**5.31** ^Ea^ ** ± **0.922
**24**							**4.19** ^Fa^ ** ± **0.637

Means with the same small letter in the same rows are not significantly different

Means with the same capital letter in the same columns are not significantly different

**Table 11:** Odor of beef burger affected by anthraquinone pigment (AQP) and γ- irradiation during cold storage (4 ± 1ºC)

Storage Period (day)	Treatments						
	**control**	**Anthraquinon concentration (%)**			**BHT (200 ppm)**	**γ- irradiation (kGy)**	
		**0.5**	**1.0**	**2.0**		**3**	**5**
**0**	**8.66**^Aa^ ** ± **0.731	**8.62** ^Aa^ ** ± **0.683	**8.53** ^Aa^ ** ± **0.503	**8.43**^Aa^ ** ± **0.636	**8.62** ^Aa^ ** ± **0.494	**8.12** ^Aa^ ** ± **0.707	**8.51** ^Aa^ ** ± **0.831
**3**	**8.56** ^Aa^ ** ± **0.747	**8.57** ^Aa^ ** ± **0.499	**8.44** ^Aa^ ** ± **0.617	**8.35** ^Aa^ ** ± **0.580	**8.55** ^Aa^ ** ± **0.497	**8.12** ^Aa^ ** ± **0.707	**8.50** ^Aa^ ** ± **0.700
**6**	**7.26**^Bb^ ** ± **0.849	**8.32** ^Aa^ ** ± **0.634	**8.32** ^Aa^ ** ± **0.790	**8.27** ^Aa^ ** ± **0.512	**8.24** ^Aa^ ** ± **0.868	**8.22** ^Aa^ ** ± **0.757	**8.42** ^Aa^ ** ± **0.503
**9**	**4.69** ^Cc^ ** ± **0.820	**7.62**^Bab^ ** ± **0.494	**7.96** ^Aab^ ** ± **0.679	**8.11** ^Aab^ ** ± **0.983	**7.43**^Bb^ ** ± **0.833	**7.82**^Aab^ ** ± **0.769	**8.26**^ABa^ ** ± **0.525
**12**		**5.71**^Cc^ ** ± **0.929	**6.23**^Bab^ ** ± **0.574	**7.81A**^a^ ** ± **0.778	**5.81**^Cc^ ** ± **0.619	**6.70** ^Bb^ ** ± **0.823	**8.10** ^ABa^ ** ± **0.994
**15**			**5.61**^Cc^ ** ± **0.690	**6.71**^Bb^ ** ± **0.664			**7.76** ^BCa^ ** ± **0.868
**18**				**5.62**^Cb^ ** ± **0.931			**7.64**^BCa^ ** ± **0.821
**21**							**7.20** ^Ca^ ** ± **0.919
**24**							**6.10** Da ** ± **0.738

Means with the same small letter in the same rows are not significantly different

Means with the same capital letter in the same columns are not significantly different

**Table 12:** Texture of beef burger affected by anthraquinone pigment (AQP) and γ- irradiation during cold storage (4 ± 1ºC)

Storage Period (day)	Treatments						
	**control**	**Anthraquinon concentration (%)**			**BHT (200 ppm)**	**γ- irradiation (kGy)**	
		**0.5**	**1.0**	**2.0**		**3**	**5**
**0**	**8.76** ^Aa^ ** ± **0.698	**8.83** ^Aa^ ** ± **0.698	**8.74** ^Aa^ ** ± **0.640	**8.68** ^Aa^ ** ± **0.700	**8.90** ^Aa^ ** ± **0.738	**8.71**^Aa^ ** ± **0.814	**8.74** ^Aa^ ** ± **0.640
**3**	**7.82**^Bb^ ** ± **0.769	**8.21**^Aab^ ** ± **0.772	**8.32** ^ABab^ ** ± **0.920	**8.51** ^Aab^ ** ± **0.831	**7.74** ^Bb^ ** ± **0.795	**8.32** ^ABab^ ** ± **0.634	**8.66** ^Aa^ ** ± **0.869
**6**	**5.31**^Cc^ ** ± **0.654	**7.23** ^Bb^ ** ± **0.998	**7.97** ^BCa^ ** ± **0.673	**8.22** ^Aa^ ** ± **0.757	**6.75** ^Cb^ ** ± **0.717	**7.91** ^Ba^ ** ± **0.725	**8.58** ^Aa^ ** ± **0.721
**9**	**3.22**^Dd^ ** ± **0.592	**5.42**^Cc^ ** ± **0.503	**7.56** ^Cb^ ** ± **0.832	**8.20** ^Aa^ ** ± **0.632	**5.18** ^Dc^ ** ± **0.643	**7.81**^Bab^ ** ± **0.792	**8.32** ^ABa^ ** ± **0.634
**12**		**4.56**^Dd^ ** ± **0.685	**5.99** ^Dc^ ** ± **0.943	**7.11**^**B**b^ ** ± **0.862	**4.14** ^Ed^ ** ± **0.490	**7.72**^Bab^ ** ± **0.655	**8.12** ^ABCa^ ** ± **0.707
**15**			**4.66** ^Ec^ ** ± **0.667	**6.30**^**C**b^ ** ± **0.949			**7.88** ^BCa^ ** ± **0.767
**18**				**5.04**^**D**b^ ** ± **0.771			**7.52** ^Ca^ ** ± **0.509
**21**							**6.26** Da ** ± **0.525
**24**							**5.68** Da ** ± **0.870

Means with the same small letter in the same rows are not significantly different

Means with the same capital letter in the same columns are not significantly different

All beef burger samples tested at zero time received high scores ranging from 8.12 to 9.30, which may be a result of their high quality and the addition of AQP at various degrees. BHT (200 ppm) and gamma irradiation (3 and 5 kGy) treatments had little to no impact on the sensory qualities.

The control sample was rejected after 9 days due to mold growth on the samples' surface and had a very low score at the conclusion of the storage period, whereas prolonged cold storage considerably and differently decreased the score for each sample. Different AQP concentrations were applied to the samples for 12, 15, or 18 days, respectively. However, beef burgers exposed to gamma radiation at dose levels of 3 and 5 kGy received a higher acceptance rating and survived for 12 and 24 days, respectively. These findings concur with those of Hawashin [28] and Moawad [29].

## Discussion

Fungi are currently thought to be the most promising sources of natural pigments, with a wide spectrum of bioactivities that will undoubtedly lead to a wide range of applications in food, pharmaceutical, cosmetic, textile and other industries. Previous research has shown that fungal-derived pigments have antibacterial, antioxidant, and anticancer properties [30]. *Talaromyces* species are among the most studied fungi due to their high pigment yield [31]. Also *Talaromyces* pigments have a wide range of biological activities, including antioxidant and antibacterial properties, and their safety efficacy has been established and is considered industrially essential in the food sector due to the absence of mycotoxin production [32]. The synthesis of AQP from *Penicillium purpurogenum* with antimicrobial and antioxidant activities was investigated in this study. The majority of anthraquinones that have been examined so far and were extracted from different sources (plants, microorganisms), showed greater antibacterial than antifungal properties. Related studies investigated the ability of *Penicillium purpurogenum* to produce pigments through a fermentation process with bioactive properties [33, 34]. The results supports the fact anthaquinone may be responsible for the antimicrobial activities. Anthraquinone-citrinin compounds are uncommon natural chemicals, according to Khamthong [35]. The sea fan-derived fungus *Penicillium citrinium* PSU-F51 produced penicillanthranin A, an anthraquinone-citrinin derivative. This substance has an equal MIC value of 16 g.mL^−1^ and moderate antibacterial activity against *Staphylococcus aureus* ATCC25923. Even though anthranin A was four times more potent than chrysophanol, it had the same inhibitory impact on *S. aureus* SK1. The naturally occurring anthraquinone derivatives 4-deoxybostrycin and nigrosporin, which were isolated from the strain *Nigrospora* sp., both exhibited inhibitory effects against *Mycobacteria*. The maximum inhibitory concentration of 15.7 M, 4-deoxybostrycin significantly inhibited various clinical multidrug-resistant *Mycobacterium* TB strains [36].

TVBN is created through the breakdown of proteins and nitrogenous substances that aren't proteins, primarily as a result of microbial activity. It has been demonstrated that both the complete microbial reduction of trimethylamine oxide (TMAO) to trimethylamine (TMA) and the microbiological deamination of amino acids are responsible for the rise in TVBN [37]. Results showed that, depending on the examined treatment, the TVBN values varied in rate as the storage period extended for all hamburger samples. This might be as a result of the inclusion of AQP, BHT, and radiation therapies, which suppress the microbes that can produce those substances. These findings closely match those of Moawad [29]. According to the unique Egyptian standard (2005), minced beef products are typically considered to be spoilt if their TVBN value is more than 20 mg N/100 g.

Malonaldehyde, a subsequent product of lipid oxidation that may give oxidized fat an unpleasant flavor, is measured by TBARS analysis. Anthraquinones can engage in redox processes and display antioxidant or pro-oxidant effects according to their identical quinonoid structures. Antioxidant and antibacterial properties of emodin and physcion have been amply proven [38]. The ortho-dihydroxy substituent in alizarin, the polyhydroxyl group at positions C1, C6, and C8, with methylation at position C3 (emodin), and the polyhydroxyl group at positions C1 and C8 with hydroxylmethylation at position C3 (aloe-emodin), are all multifunctional antioxidants that combine both chain-breaking and metal-chelating properties. This is in accordance with Yen's theory [39], on anthrones, which was published in additionally, they claimed that alizarin's strong antioxidant action may be related to its increased reducing power and metal chelating activity. On the other hand, emodin and aloe-powerful emodin's ability to scavenge hydroxyl radicals may be a factor in their antioxidant activity. Alaternin is a potentially useful and adaptable antioxidant useful for defending biological systems and functions against a variety of oxidative stresses, as evidenced by its dose-dependent inhibition of the peroxidation of linoleic acid by the thiocyanate method and its inhibitory activities in reactive oxygen- and nitrogen-mediated reactions [40]. Chrysophanol, on the other hand, hastened the peroxidation of linoleic acid.

The antioxidant capacity of anthraquinones, both natural and synthetic, and their derivatives (Emodin, aloe-emodin, alizarin, physcion, etc.), has been amply proven [38, 41–43]. They are obligated to take part in redox reactions and display antioxidant or pro-oxidant capabilities due to their quinonoid structures. According to Yen [39], the basic chemical structure of anthrones played the role of an electron acceptor, and the hydroxy substituent and methylations are multipurpose antioxidants that have the ability to break chains as well as chelate metals.

The best method for deciding whether a food product is acceptable is still sensory assessment, and it serves as the ultimate check on the product's quality. The findings of the sensory evaluation revealed that all samples had a high level of sensory acceptance when compared to the control samples. The results concerning how irradiation affects sensory qualities are similar to those from Kim [44], who found that while irradiated foods created additional volatiles and had higher TBARs, there were no appreciable variations in sensory scores between them and non-irradiated meats.

The sour off-odor, on the other hand, was the key factor that influenced the samples' refrigerated storage life, with the exception of samples that were rejected because of their bacterial count (> 1 × 10^7^ cfu/g). According to Banwart [45], the activity of Micrococcus, Lactobacillus, and Microbacterium species may be responsible for the detected sour smell. These findings suggest that these natural functional elements can be added to beef products without degrading the quality of the final product, resulting in a meat product that is healthful. According to Baón [46], beneficial additives like green tea and grape seed extracts added to cooked beef patties had no impact on sensory characteristics either. Consumers may find added value in functional benefits, but they cannot outweigh the sensory qualities of food [47].

## Conclusion

This study came to the conclusion that AQP is a powerful source with antibacterial and antioxidant effects. According to the findings, AQP (at a dosage of 2%) is a natural addition (antioxidant and antibacterial), which prolongs the shelf-life of beef burgers in comparison to controls and samples with BHT without any unpleasant sensory characteristics. Therefore, it is claimed that AQP might be utilized to extend the shelf-life of meat products, giving consumers food that contains natural additives as opposed to artificial ones, which are perceived by consumers and manufacturers as being healthier than those of synthetic origin.

## Materials and methods

### Fungal strain

*Talaromyces purpureogenus* AUMC2603 was acquired from Asuit University's Mycological Center for Culture Collection, http://www.aun.edu.eg/aumc.htm. This strain was chosen as a potential source of red pigment in this study. The stock culture was kept at 4 °C on Potato Dextrose Agar (PDA) slopes and subcultured at regular intervals for the subsequent studies*.*

### Culture condition for pigment production

Maximum pigment yield was produced under optimum condition obtained from statistical optimization methodology in our initial study [21] as follow: initial medium pH 6, temperature 25 °C for 18 day incubation period on Yeast Malt Broth (YMB) of the following composition (g/l): malt extract 3; yeast extract 3; peptone 5; glucose 10. The fermentation process was maintained under static dark condition. The un-inoculated media was used as control. These conditions were achieved using response surface methodology.

### Extraction and estimation of extracellular pigment

One-factor-at-A-time (OFAT) strategy was used to determine the best solvent for extraction of pigment. Following the end of the incubation, the biomass and fermented broth were separated by filtration using filter paper, and the supernatant containing the pigment was taken out. Different organic solvents, including diethyl ether, chloroform, hexane, n-butanol, ethanol, and ethyl acetate, were used separately, to extract the crude red pigment under shaking at 100 rpm for 24 h at 25 ± 3 °C. To get rid of the solvents, the pigment extracts were concentrated at 45C in a rotary evaporator under decreased pressure.

The extracted pigment under each extraction solvent was tested for antibacterial activity to determine the most effective extraction. Eight pathogenic bacterial species were used as test organisms for the antibacterial activity. *Bacillus cereus* GST4, *Bacillus subtilis* BW2 (obtained from Faculty of Medicine's Microbiology Department at Zagazig University in Egypt), *Escherichia coli* ATCC 11,229, *Salmonella typhi* ATCC 14,028, *Klebsiella pneumonia* ATCC 13,883, *Staphylococcus aureus* ATCC 25,923, *Pseudomonas aeruginosa* ATCC 15,442, and *Listeria inanovii* ATCC 19,119 strains (obtained from The National Research Center, Giza, Egypt). The disc diffusion method was used to assess the antibacterial activity, according to Bennett [48]. As a check, organic solvents were utilized to eliminate the solvent-related inhibition. 20 μl of dissolved extract was loaded on 5 mm sterilized disc of Whatman filter paper. Plates were then incubated at 37 °C for 24 h. Control for each organic solvent was also taken simultaneously to eliminate the solvent-related inhibition. The inhibition zone (mm) surrounding the filter was measured after the incubation. The yield of the pigment was expressed as optical density at 510 nm (OD510) of the extract which is the appropriate wavelength for maximum light absorption by produced pigment. In this study only extracellular pigments were considered.

### Pigment characterization

#### LC mass spectrometry

In this investigation, 10 mL of HPLC grade 50:50 water:ethanol (Fisher Scientific, Ontario) were used to dissolve 2.5 mg of dried crude pigments. A LC and Q-Exactive HF Quadrupole-Orbitrap mass spectrometer were used for the analyses (Thermo-Fisher, Mississauga, ON). A Kinetex 1.7 m C18 LC column (100 2.1 mm) from Phenomenex, Torrance, California, was used for the separation. Gradient elution with 95:5% water:ethanol (A) and 100% ethanol (B), unbuffered for the negative mode and 0.1 percent formic acid for the positive mode, was used to produce the separation. The column was heated to 40 °C and the solvent flow rate was 0.1 mL/min. The gradient elution started at 5 percent B, climbed linearly to 100 percent B over 20 min, held at 100 percent B for 7 min, and then re-equilibrated to 5 percent B for 8 min over the course of the 35-min run. The following source settings for positive/negative ions were used to ionise the samples: sheath gas flow was 35/30, aux gas flow was 10/8, sweep gas flow was 1, aux gas heater was 400/300 °C, spray voltage was 3.8/2.7 kV, S-lens RF was 60, and capillary temperature was 350 °C. The following scan parameters were used: 120,000 resolution, 1 × 106 AGC target, 100 ms maximum injection duration, and 100–1000 m/z entire MS scan range. Thermo Fisher's Compound Discoverer 2.1 SP1 was used to locate unidentified peaks and produce elemental compositions based on precise mass data with a 1 ppm mass tolerance. Untargeted research workflow without statistics: Find and identify unknown compounds was the built-in workflow template that was employed. It performs retention time alignment, unknown compound detection, and compound grouping across all samples. All compounds' elemental compositions are anticipated, chemical background is obscured by utilizing blank samples, and ChemSpider can be used to determine the structures of compounds (exact mass or formula). 2560 and 973 distinct features were found in the positive and negative modes, respectively. To decrease the amount of features, the post-analysis filtering listed below was carried out: 1) Peaks observed only in sample extracts and not in blanks were taken into account; Only discovered peaks with area ratios (10 ppm/100 ppm) ranging between 0.08 and 0.12 were taken into consideration. Minimum peak areas are 1 × 105.

#### Fourier transform infrared spectroscopy FTIR

Fourier transform infrared spectroscopy FTIR was used to identify and characterize pigments structure in the crude extract by using (VERTEX 80v, BRUKER, Germany) at 4 cm^−1^ resolution and measurement scale range of 4000–400 cm^−1^.

#### Pigment irradiation

Food is exposed to ionizing radiation during the food irradiation process in order to increase food safety and shelf life. In this experiment, it was also looked into how gamma radiation affected the extracted pigment. At the Nuclear Research Center in Inshas, Egypt, gamma radiation was administered. Cobalt-60 (60Co) source irradiations were carried out in a gamma irradiation cell at a dosage rate of 476.26 Gy/hr. Different irradiation dosages were used to radioactively treat pigment samples (1, 3, and 5 kGy).

### Impact of irradiation on the biological functions of pigment

#### Antimicrobial activity

The disc diffusion assay was used as reported above to test the antimicrobial activity of irradiation pigment against bacteria and fungi. On agar plates, a 24-h-old bacterial culture and a 5-day-old fungus culture were alternately distributed on the suitable cultivation medium (Nutrient agar for bacteria and PDA for fungi). Each disc was inoculated by 20 µl of irradiation pigment, and left to diffuse for 2 h at a refrigerator. Following that, the plates were incubated for 24 h for bacteria and 5 days at 37 °C for fungal strains at 25 °C. As a control, discs inoculated with the same volume of unirradiated pigment were also tested concurrently. Following incubation, the growth inhibition zones' widths were measured. For every pigment, three replicates were run against each test organism. A mean and standard deviation were used to express the data.

#### Antioxidants activity

### DPPH and ABTS radical scavenging activity

The antioxidants activity of crude pigments, crude pigments exposed to gamma rays1, gamma rays2, gamma rays3 were evaluated using DPPH and ABTS assays at different concentration (0.05, 0.01, 0.15, 0.2, 0.5 and 1 mg/ml) as previously described by [49, 50].

### DNA damage protection

To determine relevant impact of gamma ray treatment on DNA damage protection potency, the crude pigments treated with and without Gamma ray were subjected damaged DNA plasmid induces by Fenton's reagent as previously reported by Leba [51]. In brief, 2 µg/ml of each sample were added to a mixture containing 3 μl of Ribonuclease Inhibitor (RNH1) plasmid DNA (60 μg/μl), Fenton's reagent composed of 5 mM of H_2_O_2_ and 0.35 mM of FeSO_4_ and 0.60 mM of EDTA and the final volume was completed to 20 μl with phosphate buffer 8.3 mM, pH 7.4. After 20 min of incubation at room temperature. The samples were separated at 1.5% agarose gel electrophoresis % and separated bands were analyzed. Plasmide DNA treated with fenton's reagent and 3 μl of RNH1 plasmid DNA (20 μg/μl) were used as positive and negative controls.

### Cytotoxicity test

The samples were tested for their cytotoxic activity against two non-cancerous cell lines, BJ-1 skin fibroblast (ATCC® CRL-2522™) and MCF-12 epithelial breast (ATCC® CRL-10782™). The seeding, culturing and sub-culturing were carried out as previously described by [52, 53], with slightly modifications. In brief, 1 × 10^5^/well cells were plated into 100 µl of medium/well in 96-well plates (Hi media) containing different concentration of crude pigments exposed or not γ-ray at three different doses. After 48 h incubation the cell viability was detected using MTT assay as previously reported by % [54]. The concentration causes 50% inhibition of viability (IC_50_) was determined by plotting the cytotoxicity % against concentration. All experiments were performed in triplicate.

### Preparation of beef burger samples

Fresh beef meat was obtained from the local market, Zagazig city, Sharqia Governorate in the day before experiment. The meat was stored in a refrigerator at 4 ± 1 °C overnight. Burger was prepared by the common method according to Modi [55] the mixture divided into four groups as follow:

The first group was the control (no additives), the second group had 200 ppm of synthetic BHT added to it as an antioxidant, the third group was split into three portions with AQP concentrations of 0.5, 1 and 2 percent, and the fourth group was exposed to gamma radiation at dose levels of 3 and 5 kGy. All groups were formed and put into polyethylene bags before being stored at (4 ± 1 °C) and periodically analyses.

### Irradiation treatment

The irradiation treatment was carried out using a ^60^Co Russian gamma chamber (dose rate 1.3 kGy/h) belonging to Cyclotron Project, Nuclear Research Center, Atomic Energy Authority, Cairo, Egypt. The applied irradiation doses were 3 and 5 kGy for beef burger.

### Total viable bacterial count

Two identical Petri dish sets were used, together with pipette-diluted 1 ml portions of standard plate count agar (PCA, Biolife cod. No. 402145) from 10^–1^ to 10^–6^, which were then melted in the steam that followed. Agar is placed into Petri dishes after being cooled to 44–46 °C. As soon as possible, aliquots were rotated and tilted in the Petri dishes to combine with the agar medium. The Petri dishes were inverted and incubated at 37 °C for 48 h after solidification. The dilution factor was used to count and multiply the expanding aerobic colonies [56].

### Psychrophilic bacterial count

According to the American Public Health Association [56], total psychrophilic bacteria were counted using plate count media estimated using the total bacteria count method, with the exception that incubation was carried out at 7 °C for 5 days in refrigeration.

### Coliform bacterial count

In accordance with the instructions outlined in the Difco Manual, the coliform bacteria were identified using violet red bile agar media. The plates were incubated for 24 h at 37 °C [57].

### Yeasts and molds

On potato-dextrose agar medium, yeast and mold counts were determined according to the Difco Manual protocols [57]. The plates were incubated for 5 days at 20 to 25 °C.

### Total volatile basic nitrogen (TVBN)

Total volatile basic nitrogen (TVBN) was measured as follows: 50 g of the minced sample were soaked in 600 ml of distillate water for 24 h in the refrigerator. The mixture was then filtered through cotton, and the volume was increased to 1 L in a flask using distillate water. A total of 400 ml was used, followed by the addition of 30 ml of ethanol, 2 g of magnesium oxide, and 25 ml of 0.1 N sulfuric acid to obtain a final volume of 150 ml in a distillation flask. To eliminate carbon dioxide, the distillate (150 ml) was heated for 10 to 15 min. The mixture was allowed to cool at room temperature before the addition of 0.2 ml of the 0.2% rosolic acid indicator and the quick titration back of the surplus sulfuric acid with sodium hydroxide. mg TVNB per 100 g of material was used to represent the results [58].

### Thiobarbituric acid-reactive substances (TBARS)

The following values for thiobarbituric acid-reactive compounds (TBARS) were calculated using the Kirk and Sawyer method [59]. Ten grams of the sample were combined with 50 ml of water for two minutes, then were washed into a distillation flask with 47.5 ml of water. 2.5 ml of 4 M hydrochloric acid was then added to the mixture to lower the pH to 1.5, and then an antifoaming agent and some glass beads were added. The flask was heated using an electric mantle, and after 10 min of boiling, 50 °C of distillate had been obtained. A glass-stopper tube was filled with five millilitres of distillate, or five ml T.B. Glacial acetic acid at a concentration of 0.2883 g/100 ml was added, stoppered, shacked, and heated for 35 min in boiling water. In parallel, a blank was made by mixing 5 ml of distilled water with 5 ml of reagent. The tubes were then cooled in water for 10 min, and the absorbance (O.D.) against the blank at 538 nm was measured using 1 cm cells using a spectrophotometer. TBARS = O.D. × 7.8 (as mg malonaldehyde per kg sample).

### Sensory evaluation

According to the procedure outlined by Watts et al., [59], sensory evaluation of beef burger samples (untreated and treated) was done in real time and regularly every three days for their appearance, odour, texture, and general acceptability during cold storage at (41 °C) for 24 days. Ten members of the Nuclear Research Center's food irradiation team are on the panel for sensory evaluation.

### Statistical analysis

All independent analysis were carried out in triplicates (*n* = 3) for which the results were expressed as mean ± standard error. Data were analyzed using SPSS analytical software version 18.0 (SPSS Inc., Illinois, USA). Data were subjected to one-way analysis of variance (ANOVA) followed by Duncan test for comparison of means as a post-hoc test. Significant levels were based on the confidence level of 95% (*p* < 0.05). All methods were carried out in accordance with relevant guidelines.

## Data Availability

The datasets used and analyzed during the current study are available from the authors on reasonable request.
